# Pituitary protein 7B2 plasma levels in patients with liver disease: Comparisons with other hormones and neuropeptides

**DOI:** 10.3892/ol.2013.1384

**Published:** 2013-06-07

**Authors:** MARIA S. VENETIKOU, LUKE MELEAGROS, MOHHAMMAD A. GHATEI, STEPHEN R. BLOOM

**Affiliations:** 1 Department of Medical Sciences, Faculty of Health and Caring Professions, Technological Educational Institute (TEI), Athens, Greece;; 2 Department of Surgery, Middlesex Hospital, London, UK;; 3 Department of Investigative Medicine, Imperial College, Hammersmith Hospital, London, UK

**Keywords:** 7B2, pituitary protein, plasma levels, liver disease

## Abstract

7B2, a protein initially isolated from the porcine pituitary gland, has been identified in numerous animal and human tissues, with the highest concentrations in the pituitary and hypothalamus. The 7B2 molecule is highly evolutionarily conserved and is considered to be indispensable in the function and regulation of proprotein convertase 2 (PC2). In the present study, the plasma 7B2 immunoreactivity (7B2-IR) of 18 patients with liver disease was studied. Of these patients, seven (three male and four female), aged 37–67 [54.6±13.5 (SD)] years, suffered from liver cirrhosis of cryptogenic (n=2) or alcoholic (n=5) aetiology. The remaining 11 patients (four male and seven female), aged 22–76 [56.1±17.6 (SD)] years, suffered from miscellaneous liver abnormalities. The clinical diagnosis was confirmed in the majority of patients by the histological examination of a percutaneous liver biopsy or by appropriate radiological investigations. Plasma bilirubin, alkaline phosphatase, aspartate aminotransferase, albumin, prothrombin time, electrolytes, urea and creatinine were measured. The plasma 7B2-IR levels were estimated using a sensitive radioimmunoassay (RIA), and the elution position of 7B2-IR was verified by gel chromatography. The mean plasma 7B2-IR concentration in patients with liver disease was 99.44±15.9 pmol/l. In the patients with hepatocellular damage due to metastatic tumours [Ca bronchus, carcinoid (n=6)], the 7B2-IR concentrations were significantly higher [185±36.9 pmol/l, (P<0.05)] compared with the overall subjects with liver damage. The results of the present study demonstrate that 7B2-IR is increased in liver disease, with the highest levels detected in patients with tumourous liver conditions.

## Introduction

The liver is central to body metabolism and when it is affected by diseases, such as cirrhosis, its complex functions are invariably impaired. Changes in the metabolism of several hormones have been observed in liver disease.

It was reported that 8 to 68% of 44 patients with hepatocellular carcinoma had increased levels of α- and β-human chorionic gonadotropin (hCG) subunits, calcitonin, parathyroid hormone (PTH), prolactin (PRL), adrenocorticotropic hormone (ACTH) and growth hormone (GH). Due to the simultaneous increases of these hormones in cirrhosis, their production is likely to be due to the metabolic effects of liver cell dysfunction rather than ectopic production (1). Corticotrophin releasing hormone (CRH) has been shown to be secreted by NPL-KC (a human hepatoma cell line) and behave as a hypothalamic CRH (2). CRH and ACTH were observed to be produced by liver and lung metastases in a patient with a pituitary carcinoma and the clinical presentation of Cushing’s disease (3).

In males with liver cirrhosis, a reduction in free testosterone in the serum was shown to be associated with normal basal lutenising hormone (LH) and follicle-stimulating hormone (FSH), suggesting impaired function of the hypothalamic-pituitary-gonadal axis (4). The low testosterone level and the derangement of the hypothalamic-pituitary function have a role in the sexual dysfunction and changes in sex hormones that occur in male patients with cirrhosis (5). The LH levels of amenorroeic females with various aetiologies of cirrhosis were shown to have decreased below the normal range in 50% of patients with alcoholic cirrhosis and 42% of patients with non-alcoholic cirrhosis, and tests revealed that the hypothalamus, rather than the pituitary, was the site of the disturbance in gonadotrophin secretion (6). Gonadotrophin-releasing hormone (GnRH) receptors have been localised in various differentiated hepatocarcinoma tissues and their expression in these tissues is associated with the degree of differentiation (7). Treatment with a combination of sex hormone suppression and inhibition of their target receptors has been attempted in the tissues of patients with hepatocellular carcinoma (8,9).

In patients with cirrhosis, the hypothalamic-pituitary-adrenal and -gonadal axes and PRL secretion are impaired. The response of GH to GH-releasing hormone (GHRH) is also accelerated, and elevated basal and stimulated levels of GH possibly reflect compensation for the low levels of IGF-1, which is associated with deteriorating liver function (8). No effects of the aetiology of cirrhosis on the degree of alteration of the hypothalamic-pituitary glandular axes have been observed (10,11).

Serum ghrelin, tumour necrosis factor (TNF)-α and interleukin 6 (IL6) levels have been reported to be significantly higher in cirrhosis and hepatocellular carcinoma patients, whereas serum leptin levels were observed to be decreased (12). Amphiregulin (AR), a member of the epidermal growth factor family has been shown increased in liver cirrhosis and behaves as a potent pro-regenerative and survival factor (13). Neurotensin (NT) is expressed in the human fetal liver, but not in the adult; in the fibrolamellar carcinoma, (NT) is also expressed but not expressed in the regerating rat liver (14). Somatostatin (SRIH), cortistatin and SRIH receptor subtypes are present in rat Kupfer cells where they may function in an autocrine manner (15). SRIH analogs (SSTs) have produced promising results for the treatment of hepatocellular cancer and a long-acting SST analog (lanreotide; LAN) exhibited the ability to decrease the S-phase fraction, as well as induce apoptosis in HepG2 cells in a dose-dependent manner (16). Peptide 23, a newly identified protein by rat pituitary cells, is stimulated by GH and inhibited by SRIH. Peptide 23-cDNA has ∼73% homology with human hepatocellular carcinoma cDNA from human hepatocellular carcinoma (17).

The majority of liver tumours express cholecystokinin (CCK-B) receptors and are able to process gastrin (G) as far as pro-G and G-gly (precursor forms) and this may be associated with tumour proliferation (18). Gut hormone receptors are overexpressed in human cancer and allow receptor-targeted tumour imaging and therapy. A novel promising receptor for these purposes is the secretin receptor. Secretin receptors are expressed in the human liver particularly the biliary tract and cholangiocarcinomas but not the hepatocytes or hepatocellular carcinomas (19). Hepatic failure is associated with increased pancreatic glucagon levels (true hyperglucagonaemia is suppressed by glucose) (20). Methionine enkephalin and other opioid peptides have been shown to be increased in both cirrhosis and acute liver disease (21). Furthermore, there is evidence that the central mechanism involved in the brain-liver interaction in the development of symptoms of hepatic encephalopathy (such as pruritus and fatigue) involves the opioid system (22). A class of endogenous opioids is upregulated in liver disease particular to cholestasis, which contribute to pruritus, hypotension and encephalopathy. Symptoms associated with cholestasis are reversed or ameliorated by opioid receptor antagonists. Opioid receptor antagonists have been reported to relieve multiple symptoms, except for pruritus, and improve liver function as demonstrated in experimental cholestasis (23).

7B2 was identified in 1982 by Seidah *et al* during the purification of the N-terminal glycol-segment of proopiomelanocortin (POMC) from pig anterior pituitaries (24). Later, the same investigators reported the purification of its human pituitary homologue (25). The two sequences differed by only one amino acid. 7B2 cDNA cloning from various species has demonstrated the high evolutionary conservation of the 7B2 molecule, suggesting that 7B2 may be biologically relevant. In particular, the overall residue identity is extremely high (90–96%) among mammals, relatively high (67–83%) between mammals and frogs or fish, and low (17–22%) between vertebrates and invertebrates (26,27). Initially, 7B2 was revealed to be located in the pituitary gonadotrophs (26,27) and to respond to exogenous LH-releasing hormone (LHRH) (26). 7B2 has also been demonstrated to be located in tissues that are primary neuronal (the brain and adrenal medulla) or endocrine (pituitary, thyroid and pancreas) tissues, or those that are known to carry a sub-population of neuroendocrine cells (gastrointestinal tract). The highest levels are detected in the anterior lobe of the pituitary, followed by the neurointer-mediate lobe, hypothalamus, adrenal medulla, thyroid gland and pancreas (27). In cell cultures (normal pituitary cells), the secretion of 7B2 appears to be unaffected by GHRH and CRH, but is increased by LHRH (26,27).

7B2 is detectable in human plasma. It is present at remarkably high levels in early childhood, which gradually decrease to adult levels by 20 years of age and slowly rise again with aging (28,29). The levels of 7B2 are elevated in pregnancy, from the second to the fourth trimester, but sharply decline soon after delivery and return to normal by 4-6 weeks post-partum (30). Initial studies of plasma 7B2 level increases in patients suffering from chronic kidney failure and liver cirrhosis (28,29,31) have suggested that these organs are involved in 7B2 clearance.

In the present study, the plasma 7B2 immunoreactivity (7B2-IR) levels were measured in patients with liver disease, mainly cirrhosis of various aetiologies, in a further attempt to investigate the hepatic handling of this protein.

## Patients and methods

### Patients

The present study was also undertaken by Dr L. Meleagros (Endocrine Unit, Hammersmith Hospital, London, UK). In total, 18 patients with liver disease, who were under the care of Dr J. Calam (Department of Medicine, Hammersmith Hospital), were studied. The study was approved by the Ethics Committee of Hammersmith Hospital (RPMS, Imperial College London) and written informed consent was obtained by the patients. Of these patients, seven (three male and four female), aged 37–67 [54.6±13.5 (SD)] years and weighing 51–96 [69.5±17.8 (SD)] kg, suffered from liver cirrhosis of cryptogenic (n=2) or alcoholic (n=5) aetiology. The remaining 11 patients (four male and seven female), aged 22–76 [56.1±17.6 (SD)] years and weighing 50–100 [67.7±14.8 (SD)] kg, suffered from miscellaneous liver abnormalities.

The clinical diagnosis was confirmed in the majority of patients by the histological examination of a percutaneous liver biopsy or by appropriate radiological investigations (Table I). The total number of patients and the numbers with jaundice, ascites/oedema and hepatic encephalopathy are also shown in Table I.

The medication received by the patients was as follows (number of patients with cirrhosis vs. without cirrhosis): folic acid (6 vs. 0), ranitidine (5 vs. 0), prednisolone (1 vs.2), vitamin K (3 vs. 0), parentrovite (2 vs. 1), lactulose (4 vs. 0), diuretics (0 vs. 2), heparin (0 vs.1), cyclosporin A (1 vs. 0), salazopyrine (0 vs. 1), insulin (0 vs. 1), neomycin (1 vs. 0) and cyproheptadine (0 vs. 1). In addition three patients with and one without cirrhosis were on protein-restricted diets, and three cirrhotic patients were on salt-restricted diets. Peripheral venous blood samples were obtained between 08:00–09:00 hours with the patients in a seated position. All medication was withheld on the morning of the study. Samples were collected in heparinised tubes for the plasma 7B2-IR estimations and were processed as described. The plasma samples (100 *μ*l aliquots) were assayed for 7B2-IR in duplicate. Plasma bilirubin, alkaline phosphatase, aspartate aminotransferase, albumin, prothrombin time, electrolytes, urea and creatinine were measured at the Chemical Pathology Laboratory of Hammersmith Hospital.

### 7B2 radioimmunoassay (RIA)

A sensitive RIA for 7B2-IR was developed in our laboratory as described previously (32).

### Immunisation and preparation of antisera

A peptide fragment corresponding to residues 23–39 of the authentic 180 amino acid 7B2 molecule was custom synthesised (Cambridge Research Biochemicals, Cambridge, UK) and conjugated to bovine serum albumin (BSA; Sigma Chemical Co., Dorset, Poole, UK) by carbodiimide. The conjugated material was emulsified in complete Freund’s adjuvant for the primary immunisation and in incomplete adjuvant for the booster injections. Freund’s adjuvant was prepared by mixing 8.5 ml n-hexadecane (Koch-Light Laboratories Ltd.) with 1.5 ml Arlasel A (Sigma Chemical Co.) and was then completed by the addition of heat-killed mycobacteria (1 mg/ml).

Emulsified conjugate (2 ml) containing 80 pg conjugated peptide was administered to New Zealand white rabbits via 0.5 ml subcutaneous injections, one into each groin and axilla. At three months subsequent to the primary immunisation, booster injections were administered at two-monthly intervals. Each injection contained 40 *μ*g conjugated 7B2 (23–39) in 2 ml incomplete Freund’s adjuvant (i.e. without mycobacteria). The rabbits were bled from the marginal ear vein seven to ten days after each booster injection. Blood was allowed to clot at room temperature and the serum was separated by centrifugation. Each harvested serum sample was then evaluated for its ability to bind ^125^I-7B2 (23–39). For this test, 20 *μ*l undiluted serum was added to each assay tube (in duplicate) containing assay buffer and label to a total volume of 700 ml.

The antisera that exhibited >70% binding after 1 h of incubation at room temperature followed by charcoal/dextran separation were subsequently tested at three dilutions in order to determine the optimal working dilution. The antiserum used in these studies was labelled AG7 and used at a final dilution of 1/160,000, with an affinity constant of 1.9×10^11^ l/mol with respect to the synthetic fragment. The antibody was shown to cross-react by 33% with authentic porcine 7B2 on a molar basis. No cross-reactivities were observed with human insulin, proinsulin, glucagon, secretin, SRIH, human pancreatic polypeptide, ACTH, N-terminal of POMC, β-lipoprotein (LPH), β-endorphin, GH, arginine vasopressin (AVP), oxytocin, ovine corticotrophin releasing factor (oCRF) and vasoactive intestinal peptide (VIP).

### Iodination procedure

The synthetic fragment 7B2 was used for the preparation of (^125^I-Tyr4)-7B2 (23–39) by the standard chloramine T method (33).

### Standards

The standards were prepared gravimetrically using the synthetic 7B2 fragment. Aliquots (10 *μ*l), each containing 2 pmol 7B2 (23–39), were lyophilised to 10^−2^ torr and stored *in vacuo* at −20°C.

### Assay conditions

All samples were assayed in duplicate in 2-ml polysterene tubes (LKB, Luckham Ltd.). A total of 100 *μ*l of the sample was added to each assay tube. Phosphate buffer (0.4 ml of 0.6 M, pH 7.4), containing 10 mM EDTA, 7.5 mM sodium azide and 150 mM BSA was used as the assay buffer. The label and antiserum were made up in the same buffer and 100 *μ*l of each was added to each assay tube to give a final volume of 700 *μ*l.

### Separation

Subsequent to a five-day incubation at 4°C, the antibody-bound label was separated from the free label by adding 250 *μ*l of a suspension containing 4 mg charcoal (Norit GSX; Hopkin and Williams) coated with 0.4 mg clinical grade dextran (Sigma Chemical Co.) to each tube. The tubes were centrifuged at 1600 × g for 20 min at 4°C and the supernatant was aspirated immediately.

### Gamma counting

Following separation, the charcoal pellet and supernatant were counted in multi-well gamma counters (NE 1600; Nuclear Enterprises). Since synthetic 7B2 (23–39) was used as a standard, the results are expressed as 7B2 immunoreactive equivalents (7B2-IE).

7B2-IE changes per 0.9 fmol/assay tube were detected with 95% confidence limits, with intra- and inter-assay variations of <15%.

### Chromatographic profiles

Samples containing 7B2-IE were subjected to gel permeation chromatography. A 1.4×90-cm column of Sephadex G-100 was used to separate the components present in the plasma.

The column was eluted with a 0.06 M phosphate buffer (pH 7.4) containing 10 mM EDTA, 0.3% BSA and 0.2 M NaCl, at a flow rate of 3.2 ml/h at 4°C.

The column was pre-calibrated with dextran blue [molecular weight (MW), 2,000,000], horse heart cytochrome C (MW, 12,384) and a trace amount of NaI^125^. Dextran blue, cytochrome C and a trace amount of NaI^125^ were added to each sample as internal markers. The elution coefficient (Kav) for each immunoreactive peak was calculated according to the method used by Laurent and Killander (34). Gel permeation chromatography of porcine pituitary extracts showed a major peak (90% of total immunoreactivity), eluting prior to cytochrome C (32).

### Statistical analysis

Statistical analyses were performed using two-tailed t-tests. P<0.05 was used to indicate a statistically significant difference.

## Results

### Patients

The results of the liver function tests and plasma biochemistry in the patients with and without cirrhosis are presented in Table II. Among the cirrhotic patients, significantly raised plasma bilirubin and aspartate aminotransferase levels, a prolonged prothrombin time and a reduced plasma albumin level provided evidence of hepatocellular damage and impaired liver function. Among the non-cirrhotic patients, the plasma bilirubin level was slightly raised, since 2 patients in this group (one with hepatic metastases and one with cholelithiasis) had common bile duct obstruction. This group had a higher, although not statistically significant, mean alkaline phosphatase level compared with the cirrhotic group, due to intrahepatic (two patients with primary sclerosing cholangitis and one with amyloid infiltration) or extra-hepatic (one patient with hepatic metastases and one with cholelithiasis) cholestasis. However, the aspartate aminotransferase level was only marginally increased, while the plasma albumin level and prothrombin time were within or just above the normal range. Therefore, there was only minimal hepatocellular damage and impairement of liver function in the non-cirrhotic group. The plasma creatinine level was higher in the cirrhotic group compared with the non-cirrhotic group, although the difference was not significant due to the high intra-group variation.

**Figure 1.**
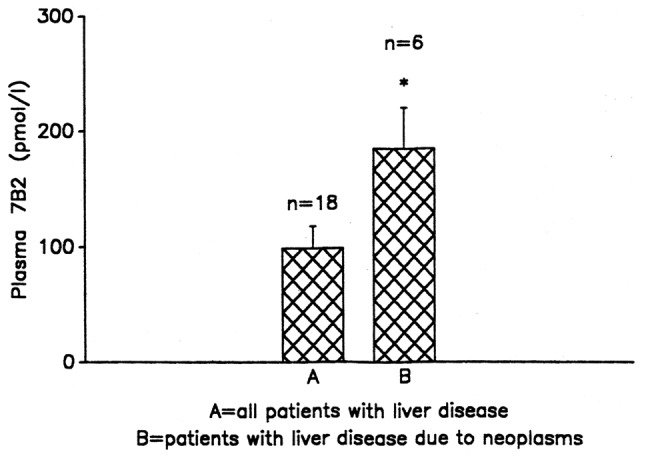
7B2 concentrations in patients with liver disease of various aetiologies compared with those observed in patients with liver disease due to metastatic tumours. ^*^P<0.05 vs. A.

The mean plasma 7B2-IR concentration in liver disease was 99.44±15.9 pmol/l. In the patients with hepatocellular damage due to metastatic tumours [Ca bronchus, carcinoid (n=6)], the 7B2-IR concentrations were higher [185±36.9 pmol/l, (P<0.05)] compared with the overall subjects with liver damage (Fig. 1).

When the 11 patients with other causes of liver damage were screened separately, the mean plasma 7B2-IR concentration was 56.68±4.5 pmol/l.

### Chromatography

**Figure 2.**
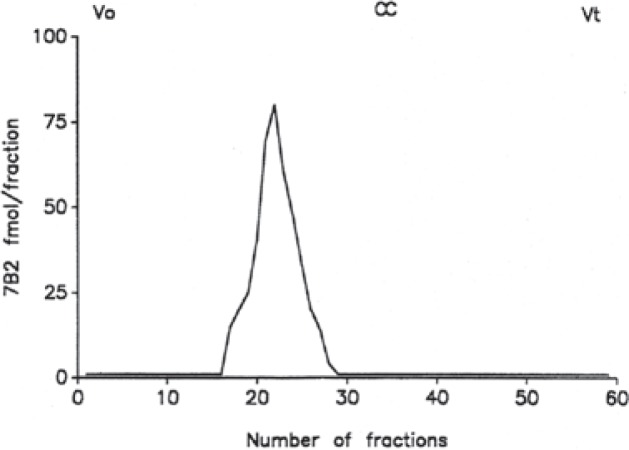
Representative plasma 7B2 chromatographic profile obtained from patients with liver disease. The column was calibrated with Vo, CC and Vt as molecular size markers. Vo, Dextran blue; CC, horse heart cytochrome C; Vt, Na 125.

Fig. 2 shows a representative 7B2 plasma chromatographic profile from the patients with liver damage.

## Discussion

In the present study, it was observed that 7B2-IR levels were increased in liver disease. In the majority of the patients with hepatocellular damage, the 7B2-IR levels ranged between 1.5- and 3-fold of the normal range compared with the control subjects. These data are in accordance with previous studies that have demonstrated increased 7B2 plasma levels in patients with cirrhosis (28). Kobori (35) also reported increased 7B2-IR in the plasma of 13 patients with cirrhosis.

In the present study, it was also evident that in individual patients with liver disease due to metastatic tumours (Ca bronchus, metastatic carcinoid), 7B2-IR was increased by >3–5-fold compared with the normal range. This agrees with the results of Suzuki *et al* (36) who showed that 7B2-IR is mainly encountered in neoplastic conditions, particularly endocrine or other tumours of the gastrointestinal tract. It is therefore difficult to conclude whether 7B2 is produced by the damaged hepatocyte or if it is released by the tumour itself, particularly for patients with liver damage due to metastatic tumours.

The immunocytochemical analysis of tumour tissues has revealed more extensive associations between 7B2 and neuroendocrine tumours than the titration of its circulating levels. 7B2 has been detected in the majority of benign, and approximately half of malignant, pancreatic tumours, particularly insulin-producing tumours (36–39). Notably, insulinomas containing only proinsulin and not the active mature form were shown to also lack 7B2 (38). 7B2-IR has been detected in bronchial carcinoids and small-cell lung carcinomas that are associated with liver metastases (36,39–41).

Various other neuropeptides have been investigated in liver diseases and alterations in their levels have been observed. Neurotensin (NT) is expressed in the human fetal liver, but not in adults. NT is also expressed in fibrolamellar carcinoma, although it is not expressed in the regenerating rat liver (42). NT has also been shown to be produced by a fibrolamellar hepatoma (14). The majority of liver tumours express cholecystokinin (CCK-B) receptors and are able to process gastrin (G) as far as pro-G and G-gly (the precursor forms), which may be associated with tumour proliferation (43,44). Gut hormone receptors are overexpressed in human cancer and allow receptor-targeted tumour imaging and therapy. A novel promising receptor for these purposes is the secretin receptor. Secretin receptors are expressed in the human liver, particularly in the biliary tract and cholangiocarcinomas, but not in hepatocytes or hepatocellular carcinomas (19). Hepatic failure is associated with increased pancreatic glucagon levels (true hyperglucagonaemia suppressed by glucose) (20). Methionine-enkephalin and other opioid peptides have been shown to be increased in cirrhosis and acute liver disease (23). Additionally, there is evidence that the central mechanism involved in the brain-liver interaction in the development of the symptoms of hepatic encephalopathy (such as pruritus and fatigue) involves the opioid system (23,44). A class of endogenous opioids is upregulated in liver disease, particularly cholestasis, which contribute to pruritus, hypotension and encephalopathy. Symptoms associated with cholestasis are reversed or at least ameliorated by mu-opioid receptor antagonists. Opioid receptor antagonists have been reported to relieve multiple symptoms, with the exception of pruritus, and improve liver function, as demonstrated in experimental cholestasis (23,44). In a multiple primary hepatic carcinoid tumour of the liver, neuron-specific enolase (NSE) and chromogranin were identified immunocytochemically in all tumour cell types. The S-100 protein, human choriogonadotrophin and serotonin were also observed (45). It has been suggested previously that 7B2-IR is co-localised with several chromogranins in the secretory granules (44,45) and that it belongs to the granin family (46). It is therefore possible that, in patients whose disease is due to primary or secondary liver neoplasms, 7B2-IR levels may be even higher than in cirrhosis of other aetiologies, due to the overproduction of 7B2 by the tumour cells, as observed in several liver neoplasms that have been positively stained for chromogranin peptides (47,48).

In neuroendocrine cells, 7B2 functions as a specific chaperone for proprotein convertase 2 (proPC2) (49). It has been proposed that 7B2 serves as an intracellular proPC2 chaperone and prevents the premature activation of the zymogen during its transit in the regulated secretory pathway. It appears that pro7B2 attaches to proPC2 in the endoplasmic reticulum (ER). This attachment is facilitated by the relatively alkaline conditions of this compartment. The inactive complex is transported to the trans-Golgi network (TGN) where pro7B2 is cleaved into an N-terminal protein and a C-terminal peptide. ProPC2 is then autocatalytically cleaved after the prodomain as the complex is transported into secretory granules. In these organelles, the prodomain and 7B2 fragments dissociate from the enzyme, which then becomes fully activated. Thus, 7B2 regulates PC2 activation (49). The 7B2 and PC2 proteins are packaged into secretory granules. It is unclear whether proPC2 requires an association with 7B2 polypeptides in order to be sorted into these organelles. Although not yet established, 7B2 may be a component of the PC2 aggregates that are sorted into the secretory granules (50,51).

Since 7B2 has been grouped with the chromogranins and secretogranins into the so-called granin family of proteins, one of its presumed functions is to facilitate the sorting of neuroendocrine proteins from the secretory granules (46). By studying POMC-expressing non-pituitary tumours, corticotrophin-like-intermediate lobe peptide (CLIP), a product of corticotrophin cleavage by PC2, was detected only in the tumours that expressed PC2 (39,51), and it may be assumed that these tumours also contain 7B2.

7B2 has also been detected in pituitary tumours (52,53). It is also noteworthy that, unlike PC2-null mice which are viable, 7B2-null mutants die early in life from Cushing’s disease due to ACTH hypersecretion by the intermediate lobe, suggesting the possible involvement of 7B2 in secretory granule formation and secretion regulation (54). The 7B2-null mice with Cushing’s disease may be saved early in life by an adrenalectomy (55). Neither our plasma ACTH tumours of Cushing’s patients (26,56), nor those studied by Natori *et al* (57,58) allow the conclusion that 7B2-IR levels in plasma are increased in Cushing’s patients, and corticotrophic adenomas have not shown increased 7B2 secretion *in vitro*, although 7B2 is concomitantly secreted with ACTH by AtT-20 tumourous cells *in vitro* (56). It has been suggested that the lethal phenotype that is exhibited by 7B2-null mice with a complex Cushing’s disease-like pathology, is due to intermediate lobe ACTH hypersecretion as a consequence of the interruption of PC2-mediated peptide processing, as well as undefined consequences of the loss of 7B2.

Sarac *et al* (59) reported that 7B2-null mice exhibit a multisystem disorder that includes severe pathoanatomical and histopathological alterations to vital organs, including the heart and spleen, but most notably the liver, in which massive steatosis and necrosis are observed. Metabolic derangements in the glucose metabolism result in glycogen and fat deposition in the liver under conditions of chronic hypoglycaemia. Liver failure is also likely to contribute to abnormalities in blood coagulation and blood chemistry, such as lactic acidosis. It has been suggested that a hypoglycaemic crisis coupled with respiratory distress and intensive internal thrombosis is likely to result in the rapid deterioration and death of the 7B2-null mice (59).

All the aforementioned observations and knowledge make 7B2 an intriguing molecule, whose definite function remains to be elucidated in view of the fact that it appears to exist in cells where PC2 activity is not evident (60), thus suggesting a bigger biological role for 7B2 compared with PC2.

The family of proprotein convertases has been implicated in tumourigenesis and metastasis in animal models. In a study assessing PC1, PC2 and 7B2 in primary colon cancers, PC1 and PC2 mRNA, protein expression and protein cleavage profiles were observed to be altered in liver colorectal metastases compared with unaffected and normal livers. Active PC1 protein was overexpressed in tumours, which correlated with its mRNA profile. Moreover, the enhanced PC2 processing pattern in the tumours was correlated with the overexpression of its specific binding protein 7B2. These results were corroborated by immunocytochemistry. The authors suggested that the exclusive presence of 7B2 in metastatic tumours may represent a new target for early diagnosis, prognosis and/or treatment (61).

Liver dysfunction may be due to various pathologies and drug interactions, and all the aforementioned observations suggest that 7B2 may be have a more biologically important role than PC2. The biological role of 7B2 appears to be important for animal survival, and more studies should be undertaken to identify a definite role for this protein, not only in liver disease, but also in other clinical entities.

## Figures and Tables

**Table I.** Characteristics of patients with liver disease.

Diagnosis	Total no.	His	Rad	Jaun	Asc/Oed	Enc
Cirrhosis	7	4	3	5	3	5
Primary sclerosing cholangitis	1	1	1	0	0	0
Hepatic metastases	6	6	6	1	1	0
Budd-Chiari syndrome	1	1	1	0	0	1
Fatty liver	1	1	1	0	0	0
Amyloid disease	1	1	0	0	0	0
Cholelithiasis	1	0	1	1	0	0

Clinical features of 18 patients with liver disease. The columns indicate the number of patients, i.e. total in each group; His, patients in whom the clinical diagnosis was confirmed histologically; Rad, patients in whom the clinical diagnosis was confirmed by radiological investigations; Jaun, patients with jaundice; Asc/Oed, patients with ascites and oedema; Enc, patients with hepatic encephalopathy.

**Table II.** Plasma biochemistry and coagulation status in patients with liver disease.

Variable	Cirrhotic	Non-cirrhotic	P-value
Bilirubin (2–14 *μ*mol/l)	203.1±81.7 (12–564)	32.4±14.8 (3–140)	<0.02
Alkaline phosphatase (30–130 IU/l)	270.9±64.3 (145–642)	477.8±125.3 (82–1062)	ns
Aspartate aminotransferase (10–35 U/l)	167.3±41.6 (38–361)	57.7±13.8 (18–98)	<0.01
Albumin (35–55 g/l)	30.3±3.1 (22–46)	38.2±1.7 (33–50)	<0.05
Prothrombin time (11–15 seconds)	20.7±2.2 (14–29)	15.2±0.5 (13–16)	<0.05
Sodium (136–149 mmol/l)	135.6±2.8 (121–142)	138.4±0.8 (134–142)	ns
Potassium (3.8–5.2 mmol/l)	3.9±0.2 (3.1–4.7)	4.3±0.2 (3.5–5.2)	ns
Bicarbonate (24–30 mmol/l)	24.1±0.8 (20–26)	25.0±1.2 (18–30)	ns
Urea (2.5–6.5 mmol/l)	7.3±2.0 (2.1–17.1)	5.3±1.2 (2.9–16.4)	ns
Creatinine (55–125 pmol/l)	148.4±46.8 (53–394)	85.9±4.3 (53–150)	ns

Liver function tests and plasma biochemistry in patients with cirrhotic (n=7) and non-cirrhotic (n=11) liver disease. Normal ranges and units are in brackets in the variable column. Results are expressed as the mean ± SEM with the range in brackets. P-value shows the significance of differences between the groups. ns, not significant.
